# Gasification reactor engineering approach to understanding the formation of biochar properties

**DOI:** 10.1098/rspa.2015.0841

**Published:** 2016-08

**Authors:** Andrew N. Rollinson

**Affiliations:** University of Nottingham, Energy Technologies Building, Innovation Park, Triumph Road, Nottingham NG7 2TU, UK

**Keywords:** gasification, polycyclic aromatic hydrocarbon, biochar, renewable energy, biomass

## Abstract

The correlation between thermochemical provenance and biochar functionality is poorly understood. To this end, operational reactor temperatures (spanning the reduction zone), pressure and product gas composition measurements were obtained from a downdraft gasifier and compared against elemental composition, surface morphology and polyaromatic hydrocarbon content (PAH) of the char produced. Pine feedstock moisture with values of 7% and 17% was the experimental variable. Moderately high steady-state temperatures were observed inside the reactor, with a *ca* 50°C difference in how the gasifier operated between the two feedstock types. Both chars exhibited surface properties comparable to activated carbon, but the relatively small differences in temperature caused significant variations in biochar surface area and morphology: micropore area 584 against 360 m^2^ g^−1^, and micropore volume 0.287 against 0.172 cm^3^ g^−1^. Differences in char extractable PAH content were also observed, with higher concentrations (187 µg g^−1^ ± 18 compared with 89 ± 19 µg g^−1^ Σ16EPA PAH) when the gasifier was operated with higher moisture content feedstock. It is recommended that greater detail on operational conditions during biochar production should be incorporated to future biochar characterization research as a consequence of these results.

## 1. Introduction

How to engineer biochar such that it can provide long-lasting soil fertility (as found in ancient dark earths such as the Amazonian *terra preta do indio*) remains an intriguing puzzle. High crop yields and prolonged fertility do not occur by simply applying charcoal from slash and burn agriculture to soil [1,2]. Owing to the interdisciplinary nature of the subject, previous research has given insufficient focus to char production methods. Consequently, experiments with charred biomass produced under varying conditions of residence time, temperature, feedstock type and heating rate have resulted in inconclusive or weakly resolved findings [1–6]. It is now acknowledged that ‘the effect of biochar and pyrogenic C on soil cannot be generalized and a closer examination should be taken of biochar production processes’ [1]. However, this has yet to be fully reflected in research literature.

All charcoal is created by pyrolysis, which occurs during combustion (natural and industrial) and gasification [7,8]. The techniques used were practised initially with wood piles but more recently in kilns and retorts [7]. During pyrolysis, the biomass splits into a volatile component ‘pyrolysis gas’ (*ca* 80% by volume) and a fixed carbon (FC) framework containing inert minerals (*ca* 20%)—the ‘char’ [9]. When char from any of these processes is considered for use as a soil improver, modern terminology defines it as ‘biochar’.

Evidence points to two properties of biochar that seem most important: surface morphology, which is believed to influence water retention and encourage soil biota [1,10,11]; and the presence of polycyclic aromatic/polyaromatic hydrocarbons (PAHs)—in particular, the degree of polymerization of the carbon matrix—which gives the biochar longevity and facilitates nutrient retention [12]. Surface morphology has been shown to be a function of pyrolysis temperature and residence time [10,13–15], whereas PAHs are inherently formed during biomass combustion, pyrolysis and gasification. PAH functionality changes significantly owing to the chemical, thermal and temporal conditions to which they are exposed, constituting both the FC framework and the volatile component of pyrolysis [14,16]. In situations where the volatile hydrocarbons are not immediately purged, this can lead to PAH adsorption (not just PAH-derived secondary char deposition) within the FC framework [17]. Owing to the carcinogenic nature of some of these PAH species, there is a concern that applying biochar to soil could pose some risk to the food chain [1]. Yet, there is uncertainty as to their bioavailability [18], and tests to assess for any influence on plant growth have so far been inconsistent or inconclusive [4,5].

In chemical engineering, the formation of volatile PAHs from biomass pyrolysis and gasification (in engineering parlance ‘tar’) has been extensively studied over the last 30 years because these condensable molecules become a process line contaminant [19–22]. Research has shown that dry biomass composition has little influence on the type of tar molecules produced: temperature and reactor type are the over-riding factors, with to a lesser extent residence time [16]. Yet, and likely owing to the cross-disciplinary nature of the science involved, most analytical research done by the soil science community continues to assess the contribution of feedstock species to biochar PAH content, an oversight highlighted by a recent review [18].

The first group of long-chain hydrocarbons that evolve from simple biomass pyrolysis at moderate temperatures are categorized as ‘primary’ tars. These are relatively low-weight (C_2_–C_8_) molecules such as levoglucosan, hydroxacetaldehydes, furfurals and methoxyphenols [20]. Simple low-temperature pyrolysis will generate predominantly this group, along with some ‘secondary tar’ molecules [23]. Secondary tar is synthesized from the primary pyrolysis products, particularly above 500°C, and contains (generally C_5_–C_18_) phenolics, olefins and aromatics [20,23]. With heightened temperatures (above *ca* 800°C) and residence times, another class of tars form owing to polymerization of primary and secondary tars [24]. These ‘tertiary’ tars are subdivided into PAHs without oxygen substituents (generally C_6_–C_24_) such as ‘condensed tertiary’: naphthalene, acenaphthylene, anthracene/phenanthrene, pyrene and ‘alkalized tertiary’: methylacenaphthylene, methylnaphthalene and indene [20,23].

In older studies on biochar properties, it was not uncommon for researchers to merely describe their char samples as having been ‘produced by local farmers’ [25]. There has been some change since then, but even where the char has been produced in-house, and/or the gasifier/pyrolyser is described, detail sufficient to draw conclusions on PAH formation (e.g. actual core temperature and reactor stability during experiments) is invariably omitted [6,18,26–32]. This applies across all scales from low- to high-tech industrial systems [18], and the situation goes some way to explaining why PAH concentrations in biochar have been observed to range from 0 ≤ µg g^−1^ ≤ 3000. This also makes it difficult to draw rigorous comparisons between contemporary results. Yet, glimpses of a pattern appear: that char produced by gasification (and not just high-temperature conditions) results in a higher biochar PAH content [1,5,18,27,28]. Studies of characterized gasification biochar are particularly scant however. Of these, none reported on the monitoring of important internal reactor parameters by which PAH formation can be inferred (table 1).

**Table 1.** Extent of detail given to production conditions in previous academic research to assess PAHs in gasification biochar.

	description of char production methodology with respect to gasifier	PAH concentration (μg g^−1^) Σ16EPA unless otherwise stated	reference
	‘Char has a residence time in the fluidized bed and thermal cracker (which comprises gasification) of 12.5 s’. System not described further	45 µg g^−1^ gasifier char	[27]
	no detail of gasifier type or conditions of production are given other than a statement of ‘production temperature at 845/850°C’	255.3 µg g^−1^ gasifier char	[5]
	system (a rice husk gasifier) not identified, but known to this author as an Ankur [see description in 36]. No experimental section on how char was produced, and no information given on operational conditions	15 ≤ µg g^−1^ ≤ 115	[29]
	one sentence that describes ‘Different wood gasifiers…with process temperatures up to 1200°C’. Note that elsewhere a contradictory value of 800°C HTT is given	3000 µg g^−1^ ± 1500 µg g^−1^	[1]
	named supplier external system described as ‘Test gasifier plant operated under negative pressure at around 1000°C, the pyrolysis section around 500°C and the drying zone at 200°C’	8.7 µg g^−1^	[28]
	char ‘purchased from external vendors’, and a single stated temperature of ‘520°C. No other detail given on production conditions or gasifier system. Note that 520°C is well outside normal gasifier operating range	zero detectable. A novel methodology of subjecting the chars to overnight heating and then 3 h of leaching prior to analysis, likely explains this unprecedented value	[31]
	system described only as ‘air gasification’ in a ‘multistage updraft moving bed reactor’. Elsewhere it is stated that the char left the ‘gasification chamber at 450°C’	52.44 µg g^−1^. Additional (non Σ16EPA) PAHs were also measured, with total quantity reported as 117 µg g^−1^	[30]
	‘Bench-scale gasifier operated at approximately 800°C’. No other information provided	117 ≤ µg g^−1^ ≤ 172. Additionally, 48 ≤ µg g^−1^ ≤ 53 reported for combined quantity of naphthalene 1- and 2-methyl	[32]
	two different gasifier system used and mode of operation described in detail. However, only a range of design operating temperatures given rather than results of temperature during actual production	0.69 ≤ µg g^−1^ ≤ 5. This reports only nine of the sixteen EPA PAHs	[33]
	char sourced from an unknown external gasifier system. Design range of 600°C to 900°C	321 ≤ µg g^−1^	[6] (EAN Marks 2015, personal communication)

Exacerbating the problem even further is the fact that many variations in post-production sample preparation are used. Recently, standard methods have been proposed for biochar characterization, but prior to this a variety of solvents and extraction methods were used to obtain evidence of PAH content, giving rise to a broad spread of results [18,34]. Notwithstanding this, many studies quantify the extractable PAH content in biochar by reporting on a select range of 16 molecules named by the US Environment Protection Agency as ‘priority pollutants’ (Σ16EPA PAH). Of those which do not, their analyses are often applied to an abridged list of the same group [18,34], and frequently no validation is given for abridgement. Studies on other extractable PAHs such as heterocyclics, furans or aliphatics are often omitted, likely owing to their being no definite conclusions about the significance of these molecules.

In addition to the different types of gasifier, variables such as feedstock moisture and power output also have a major influence on internal temperature and thence gas, char and tar composition [35,36]. The downdraft gasifier design, originating in the 1920s, has been most widely used for small-scale off-grid power applications [8,37], and this type of gasifier has stratified temperature zones (feedstock drying, then pyrolysis, followed by high-temperature combustion and reduction) through which the evolved pyrolysis gases pass. The reduction zone comprises product char, and the major reactions that occur therein are (R1–R3). This feature increases the propensity for greater PAH occlusion or surface chemical interaction with the biochar, in addition to the synthesis of higher weight PAH molecules. For a more thorough explanation of gasifier design and thermochemistry, see [8,36,38].

R1 $$\begin{array}{r} C+CO_{2}\to 2\mathrm{CO}\;(\text{Boudouard reaction})\;\;\;\Delta H=+172\;\mathrm{KJ}\;\mathrm{mo}l^{-1}, \end{array}$$

R2 $$\begin{array}{r} C+H_{2}O\to \mathrm{CO}+H_{2}\;(\text{water gas reaction})\;\;\Delta H=131\;\mathrm{KJ}\;\mathrm{mo}l^{-1} \end{array}$$

R3 $$\begin{array}{r} \mathrm{and}\;\;\mathrm{CO}+H_{2}O↔CO_{2}+H_{2}\;(\text{water gas shift reaction})\;\Delta H=-41\;\mathrm{KJ}\;{\mathrm{mol}}^{-1}. \end{array}$$

It is not suggested that the people who created char for ancient dark-earth soils such as the *terra preta* will have operated a gasifier. Gasification technology was developed in the nineteenth century [38]. There are however comparisons between these ancient and modern methods of producing biochar. It is known that many indigenous communities maintain constantly slow burning low heat output fires [39]. Such prolonged incomplete combustion methods are akin to the slow heating of many industrial pyrolysis retorts and also downdraft gasifiers, exposing the biomass fuel to high temperature along with creating the conditions where volatile gases can interact with the residual char as the fire smoulders. Contrast this with some research methodologies frequently used to elucidate biochar formation such as thermogravimetric analysis (TGA), where the volatile hydrocarbons that comprise extractable PAHs are removed as they form. Indeed, TGA systems are often operated specifically to render gas transfer effects negligible by removing both evolved and purge gases from the sample as quickly as possible [40]. This explains why extremely low values of PAH concentration in biochar accompany purged reactor or vacuum studies [17] and why the largest values obtained for PAH in biochar are those derived from wood gasifiers, field trials or other traditional techniques [18].

Therefore, the aim of this study was to elucidate how biochar properties are affected and can be explained by the reactor conditions that pertain during closely monitored production. A multidisciplinary approach was taken such that in addition to real-time gasification monitoring, the biochar was then characterized using newly devised standard procedures. This characterization was focused on key properties that have been identified as most relevant from a soil science perspective.

## 2. Method

The variable in these gasification/biochar production experiments was feedstock moisture content. A 0.5 m^3^ batch of Pine (*Pinus* ssp.) wood chip was acquired from a local arborist in the Midlands region of England in the form of whole tree (heartwood and bark, but not leaves) chippings. These had been obtained from a rotating drum chipper to a standard compliant with European specification P45 [41]. Seasonal time of felling was unknown.

During preliminary gasifier experiments, it was found that additional screening of as supplied European P45 standard feedstock was necessary to remove fines, and for this, a 7 mm cut sieve was used. Without feedstock screening, pressure drop gradually increased across the reactor bed whereupon only 1 day of operation could be achieved lest a complete clean out of the reactor was necessary. Overly large or elongate pieces were also removed during the screening process as these were found to block the auger.

The screened batch of wood chip was then randomly separated, under cover, into two lots of 0.159 m^3^ (sufficient to fill the gasifier hopper). Each lot was then subdivided into 20 sublots and from each of these sublots, a value of moisture was obtained. Moisture was measured using a Dusiel digital moisture meter (probe) model MD812. Moisture was measured immediately prior to the gasification experiment to avoid the introduction of any bias from subsequent atmospheric moisture losses (or gain). Using passive drying techniques on the second lot, two different moisture levels were prepared: 7% and 17%. Both these moisture levels are achievable by passive drying, relate directly to practical applications, and were within the suggested system tolerance range of an Imbert-style downdraft gasifier [36,38]. Hereafter, these two experimental conditions will be referred to as ‘FM7%’ and ‘FM17%’. Moisture datasets are provided in the electronic supplementary material.

### (a) Gasifier system

**Figure 1.**
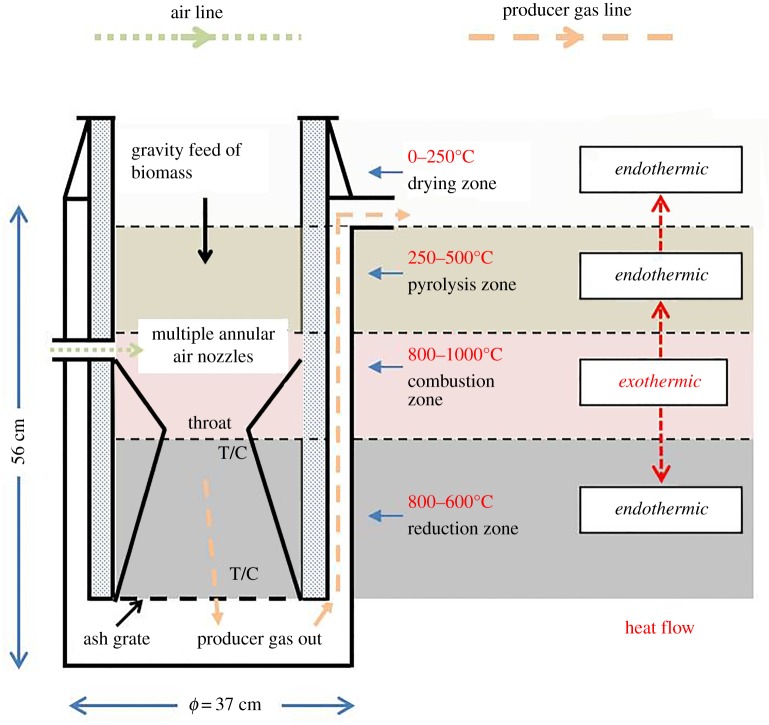
Schematic of downdraft gasifier reactor used for char production showing energy transfer mechanisms and thermal stratification. Distance between thermocouples is 15 cm. (Online version in colour.)

**Figure 2.**
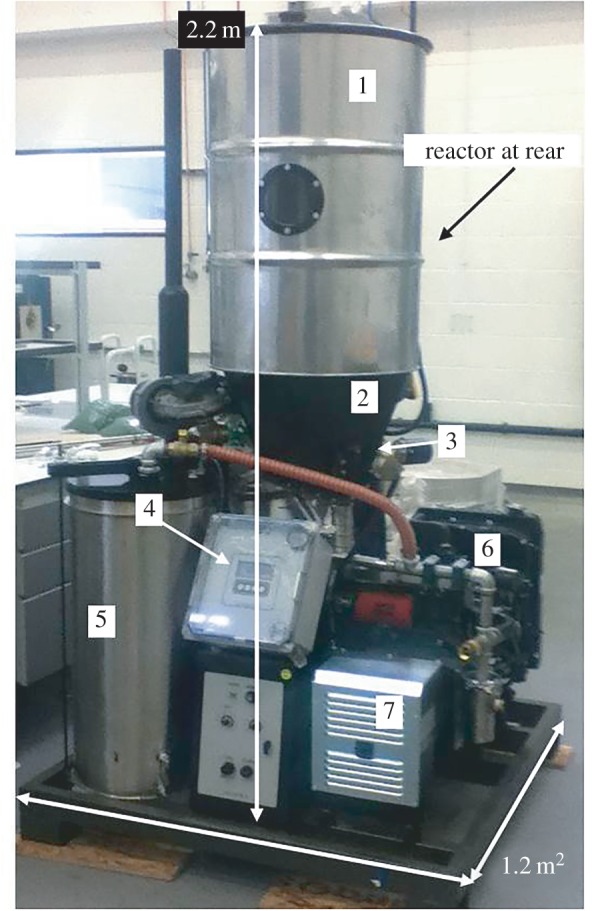
The power pallet, Mk 4. 1, feedstock hopper. 2, auger channel heat exchanger. 3, auger feeder. 4, electronic control system. 5, gas filter. 6, engine. 7, generator. (Online version in colour.)

A 10 kW power pallet gasifier from GEK All Power Labs (USA) was used for experimentation (figures 1 and 2). This comprised a conventional Imbert-style downdraft reactor combined with proprietary controls. The system was designed for small-scale off-grid electricity production from wood chips within the 1.3 ≤ cm ≤ 3.8 range and with less than 10% fines (small pieces and dust) reported to be tolerable. The feedstock, once loaded into the hopper, was fed to the reactor by an automated 7 cm diameter smart auger, activated using an internal fuel-level paddle switch sensor. All gasifiers of this classic design operate under slight negative pressure created during steady-state operation. In this case, the suction was provided by a three-cylinder Kubota gas spark ignition engine (although at start-up and shutdown, electric fan blowers were used to divert dirty gases from the engine to a flare stack via a manually operated valve). The producer gas was cleaned and passively cooled prior to the engine by passing through a cyclone and then a dry filter system comprising feedstock quality wood chips, wood chip screenings (fines) and two oiled foam filters of 5 cm thickness. A bespoke distribution box was built (Distribution Zone, UK) and through this the electrical load from the gasifier generator was dumped into resisters of 4 kW_e_ combined rated capacity (Cressall, UK). Duration of each gasification experiment was 6 h. Temperature and pressure were measured by two K-type thermocouples positioned inside the gasifier reactor, vertically 15 cm apart, in contact with the charred feedstock at either end of the reduction zone (figure 1). Real-time readouts of temperature from both measurement points, along with pressure, were provided by the system. Further details on the system configuration can be found at [42].

Preparatory to the gasification experiments, the reactor had been emptied and then filled with lumpwood charcoal in the size range of 1.5 ≤ cm ≤ 5 (from Big Green Egg, UK) up to 10 cm above the throat (figures 1 and 2). It was then loaded with the virgin woodchip (as §2) and run for approximately 6 h. During operation, residual char diminished in size and collected below the reactor's supporting grate (shown in figure 1). All future char was then assumed to originate from the feedstock.

After the filter system and prior to the engine, the producer gas was sampled exactly one and a half hours into the 6 h run. Samples were taken every 5 s, using an online Gasboard 3100P analyser by Wohun Cubic with inbuilt pump and adjustable flow meter set at 1 l min^−1^. Non-destructive analytical cells determined CO, CO_2_, CH_4_ and C_n_H_m_ by dual beam NDIR, H_2_ by thermal conductivity and O_2_ by proprietary electrochemistry. The analyser was equipped with a preliminary gas cleaning kit comprising a water trap (filled with 350 ml of room temperature tap water), carbon filter and finally a 0.3 µm polypropylene fibre filter (F3000 by CDK). The analyser was calibrated using high purity bottled gases by STG, UK.

After shutdown of the gasifier, the reactor was left to cool for 15 h before the char was removed from the ash grate receptacle. It had therefore been allowed to cool passively without exposure to ambient air or quenching. Once removed, the char was stored in brown glass screw-topped jars, in dark cool conditions.

### (b) Characterization

In compliance with the European Biochar Certificate [43], random 30 ml aliquots were arbitrarily gathered from each batch of gasifier char. The char was already in the form of small fragments (size range of fine dust to less than or equal to 7 mm); therefore, no further size diminution was undertaken in accordance with the recommendations of Hilber *et al*. [44].

The gasifier feedstock (virgin wood chip) samples were also characterized, and for this, size reduction was necessary. A Retch ZM200 centrifugal rotor mill with a 0.50 mm cut stainless steel ring sieve was used to produce a particle size of 80% less than 0.25 mm.

#### (i) Proximate and ultimate analysis

A TA instruments Q600 thermogravimetric analyser was used to ascertain proximate values. Experimentation was in triplicate, at constant 1 bar pressure using high purity (99.98%) N_2_ and air, both from Air Products UK. Data were logged every 0.8 s and saved on a personal computer. From room temperature, the samples (5 ≤ mg ≤ 10) were placed in a ceramic pan and heated to 110°C under N_2_ flow (of 40 ml min^−1^) for 2 min, followed by a hold time of 10 min. Temperature was increased to 900°C at a rate of 50°C min^−1^, and then held for 20 min. The carrier gas was ultimately switched to air and held for 20 min.

For ultimate analysis, samples and standards were weighed (70.1 ≤ mg ≤ 73.8), placed into tin capsules then inserted into a LECO CHN628. Experimentation was also in triplicate. Separate non-dispersive infrared (NDIR) cells detected H_2_O and CO_2_, with NO*_x_* passed through a tube filled with copper to reduce the gases to N_2_ and remove any excess oxygen present from the combustion process. All gases passed through LECOSORB and Anhydrone to remove CO_2_ and water before entering a thermal conductivity cell to detect N_2_. The standard used was 2,5-bis,5-(tet-butyl-2-benzo-oxazol-2-yl)thiophene.

#### (ii) Surface area, pore size and volume

Char samples (0.23 ≤ g ≤ 0.29) were analysed (in triplicate) for surface area, and pore structure by the nitrogen absorption technique with a Micromeritics ASAP 2420. High purity helium was used as carrier gas and nitrogen as adsorbate for a range of N_2_ partial pressures from 0.00 to 1.00. The Brunauer–Emmett–Teller method was used to determine the char sample surface area, and the Harkins–Jura *t*-plot method was used to estimate micropore area and volume [45,46].

#### (iii) Soxhlet extraction of polyaromatic hydrocarbons from char

Char samples (2.0 ≤ g ≤ 2.1) were inserted into 22Ø × 80 mm ceramic thimbles (Fisherbrand), lightly covered with cotton wool, and then subjected to soxhlet extraction for 36 h using 150 ml of 100% toluene [43,44,47]. After completion, the sample volume was reduced by gentle rotary evaporation using a Buchi R-240 set at 77°C and 0.5 r.p.m., followed, where necessary, by low velocity nitrogen blowdown. No additional clean-up procedures were employed, as per the recommendations of Hilber *et al.* [44].

#### (iv) GC–MS

An Agilent 7890B gas chromatograph, interfaced to an Agilent 5977 mass spectrometer was operated at full scan mode (*m*/*z* 50–450). Separation was achieved on a HP-5MS 5% phenyl–methyl silox capillary column (30 m × 0.25 mm i.d. × 0.25 µm), with helium as the carrier gas, and an oven programme of 50°C (hold for 2 min) to 300°C (hold for 33 min) at 4°C min^−1^. Spectral peaks were identified, using the NIST library [48]. The abundance of each individual PAH molecule was quantified by comparison of its peak area to that of an internal standard: 1–1 binapthyl (Acros Organics), assuming a response factor for each compound of 1 : 1. A preliminary run was completed to set the standard concentration in a comparable range to that of quantities of PAH in the sample. As a wide range of concentrations were detected, for increased accuracy, experiments were repeated using an additional one-twentieth sample dilution.

## 3. Results

### (a) Biochar production conditions

**Figure 3.**
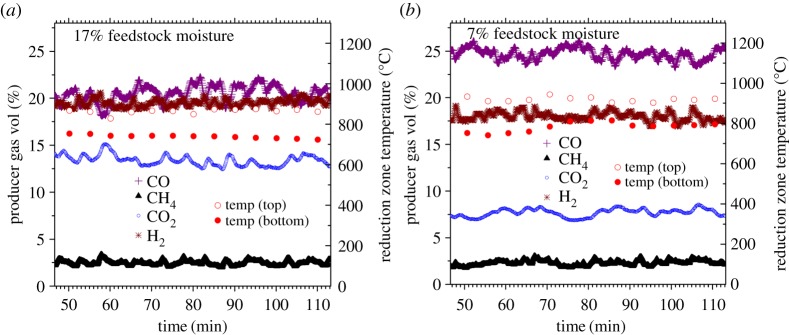
Gas composition and reduction zone temperature during steady-state operation with feedstock of (*a*) 17% moisture, and (*b*) 7% moisture. Ttred, top of reduction zone; Tbred, bottom of reduction zone. (Online version in colour.)

Figure 3 illustrates the gasification output during char production experiments with FM17% and FM7%. These are representative outputs, as duration of product gas data-logging was limited by the memory size of the gas analyser. Not shown are the C_n_H_m_ concentrations that were measured at less than or equal to 8 ppm throughout and so considered negligible. Temperature and pressure were logged every 5 min for the full 6 h of gasification.

As can be seen from figure 3, the higher moisture feedstock (FM17%) created a lower reduction zone temperature in comparison with the drier feedstock (FM7%). Table 2 shows the thermal stability of the reactor over the full duration of each 6 h run, with average values differing by 65°C at the top of the reduction zone and 49°C at the bottom of the reduction zone over the 6 h run. Full data on these extended temperature and pressure measurements are provided as the electronic supplementary material. Significant differences in gas composition and calorific value occurred as a consequence. During the gas sampling period, 21% less CO and 84% more CO_2_ concentration was detected at steady state when the system was run with FM17% compared with when FM7% was used. This subsequently decreased the producer gas gross calorific value (calculated from the sum of gaseous components) from 5.89 ± 0.1 with FM7%, to 5.47 ± 0.2 MJ m^3^ with FM17%.

**Table 2.** Mean average gasifier operating conditions throughout 6 h of operation (samples recorded every 5 min). Values in the parentheses indicate standard deviation about the mean.

	FM7%	FM17%
gasifier operation conditions		
top of reduction zone temp. (°C)	916 (23)	862 (21)
bottom of reduction zone temp. (°C)	770 (28)	731 (9)
* P*_ratio_	33 (4)	37 (4)

There was a slightly higher pressure drop across the reactor when operating with FM7%, evidenced by the lower value of *P*_ratio_, although standard deviation was identical. These pressure differential values are based on manometer water column readings across the reactor so are system-specific and have no SI units. Both were in the normal operating range of the gasifier. As a guideline value, a clean out is usually required when *P*_ratio_ is constantly below *ca* 26.

### (b) Char characterization

The char removed from the base of the reactor had fine particulates that readily became airborne from the batch, forming a fine mist under laboratory handling. Respiratory protection measures were therefore necessary for operator safety [49]. The bulk density of the char was 0.17 g cm**^−^**^3^, and *ca* 1.5 l were created per each 6 h run.

Table 3 illustrates the elemental and proximate compositions of each char sample in comparison with the gasifier feedstock. As expected, FC predominated in both chars, but there were significant differences in relative quantities with 26% more FC in the FM7% char. There were no significant differences in elemental hydrogen and nitrogen. Ash content was relatively higher in the FM17% sample, likely evidencing sample heterogeneity.

**Table 3.** Elemental values are on a dry ash free basis. Proximate values are on a dry basis. All values are wt%. Standard deviation values in parentheses.

	FM7%	FM17%	feedstock
N	0.71 (0.02)	0.64 (0.09)	0.29 (0.02)
C	81.68 (0.96)	71.37 (7.21)	50.68 (0.05)
H	0.28 (0.15)	0.56 (0.12)	6.11 (0.03)
O	17.33 (1.09)	29.77 (7.47)	42.92 (0.07)
volatile carbon	9.63 (1.41)	15.34 (0.87)	81.73 (0.75)
fixed carbon	77.01 (3.75)	60.95 (4.54)	16.47 (0.37)
ash	13.29 (2.34)	23.68 (4.34)	1.80 (0.38)

**Figure 4.**
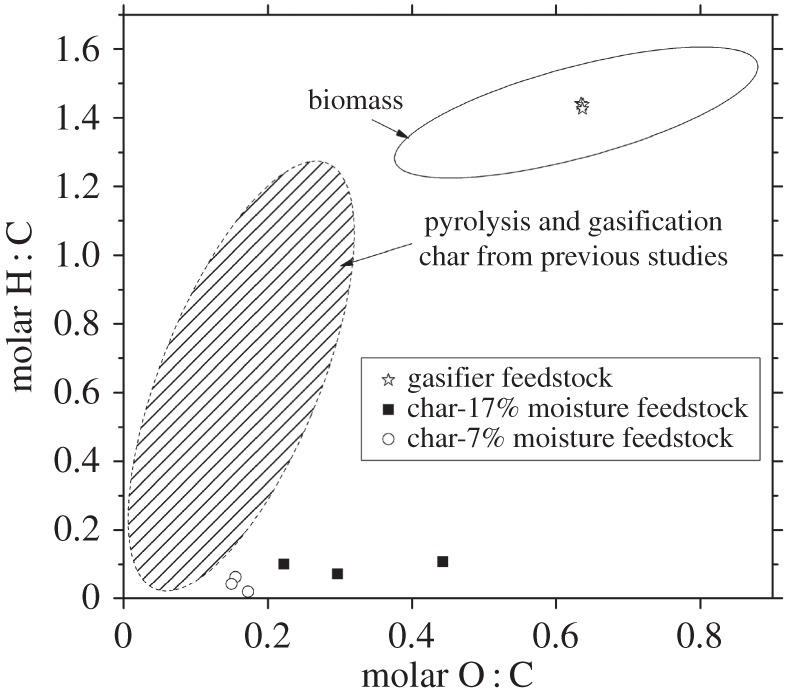
van Krevelen diagram [50] shows molar ratios of oxygen and hydrogen relative to carbon in the char. Region assigned to biomass from [1,6,14,30,33,34,51]. Note that for the gasifier chars in [1] there were also three excluded outliers (out of a total of eight samples) with O : C > 0.8, and varying H : C up to 1.3.

Although the elemental ratio values for gasifier feedstock plot within the range recorded from other studies, the char H : C and O : C ratios do not (figure 4). It must be noted however that the range of literature values for wood char is very diverse, reflecting the weak standardization in this field. Both FM17% and FM7% chars had comparable H : C ratios. But, although the ratio O : C for FM7% char was tightly constrained, the same parameter for FM17% char was detected over a much wider spread. When comparing these results with the proximate analyses (table 3), it can be seen that the wider range of elemental values in the FM17% char underlie this phenomenon.

Large differences were detected in surface morphology between FM17% and FM7% char samples. Table 4 shows how the surface area of the char exhibited a 60% increase when produced from the FM7% sample. There were also equally significant increases in micropore area (by 62%), and micropore volume (by 67%) when the drier feedstock was gasified. Of note again are the higher standard deviation in results from the FM17% char samples.

**Table 4.** Results of char surface area characterization. Standard deviation values in parentheses.

post-gasification char	FM7%	FM17%
bet surface area (m^2 ^g^−1^)	748.5 (7.7)	467.1 (50.3)
*t*-plot micropore area (m^2 ^g^−1^)	584.0 (2.8)	359.8 (13.3)
*t*-plot micropore volume (cm^3 ^g^−1^)	0.287 (0.001)	0.172 (0.015)

### (c) GC–MS

There was 94% and 86% qualitative repeatability in the GC–MS results for extractable hydrocarbons from FM17% and FM7% chars, respectively. There was 100% repeatability across all char samples for the Σ16EPA PAHs, although only those up to benzo[a]anthracene were detected (table 5). The largest concentration of extractable molecules were relatively low molecular weight benzaldehyde, benzylalcohol (although both absent in one FM7% replicate) and bibenzyl. No PAH molecule with more than four rings was detected in any sample.

**Table 5.** Molecules determined by GC–MS analysis of char extractate. Average ratios given where species identifiable in all four samples. EPA16 molecules are identified by grey shading.

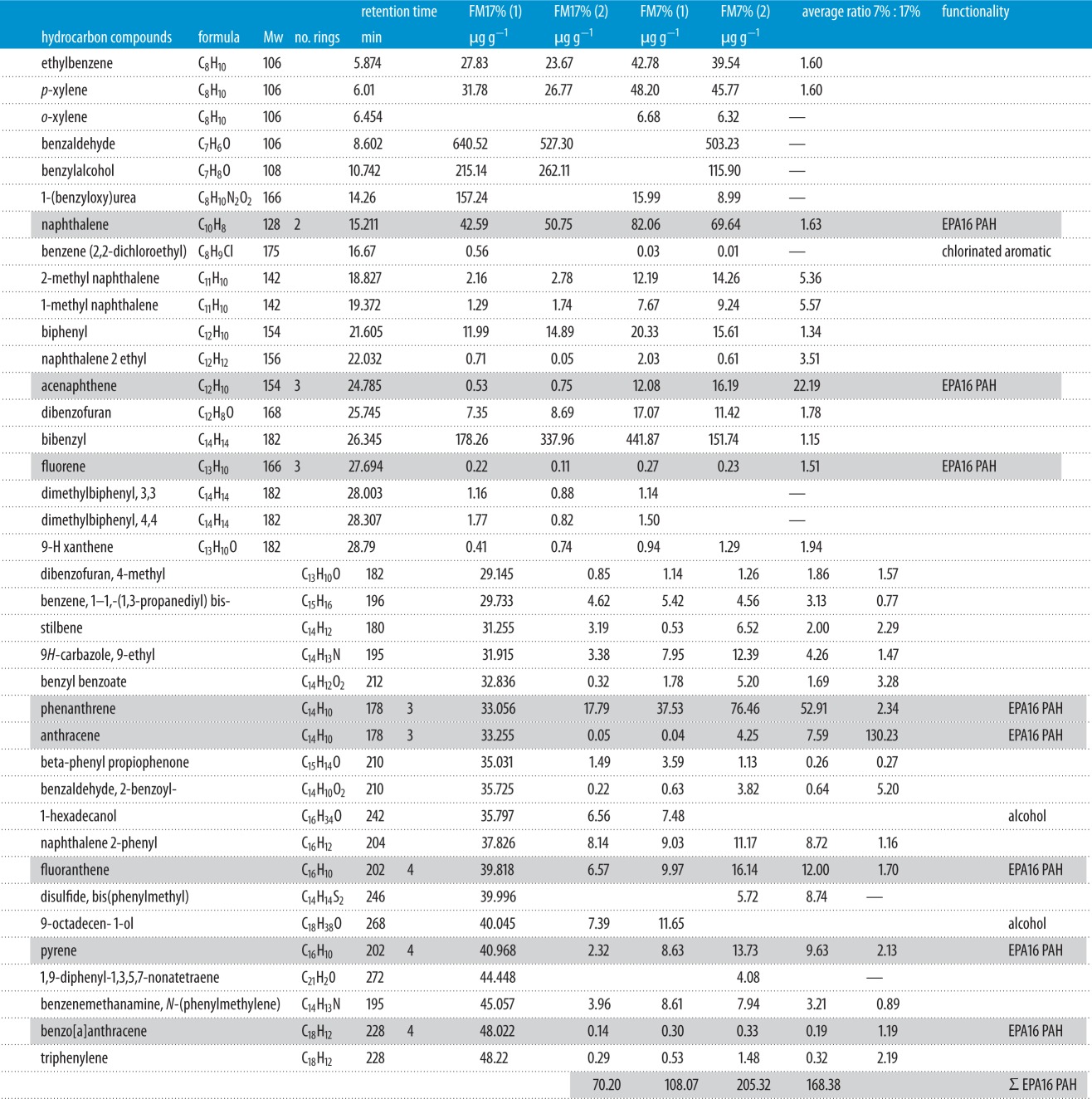

A clear dependence on feedstock moisture content and char extractable PAH was observed, with a higher concentration of PAHs in the FM7% char. Total concentration of Σ16EPA PAHs was 89 ± 19 for FM17% and 187 µg g^−1^ ± 18 for FM7%. Average ratios between the two different operating conditions (FM7% : FM17%) were calculated where a species appeared in all four samples, as shown in table 5. Of particular note were the differences in acenaphthene and anthracene between the two different chars, with concentration ratios of 22 : 1 and 130 : 1 (FM7% : FM17%).

Some trends in the degree of polymerization were also evident in the extractable PAHs. Two aliphatic molecules were only present in extract from the FM17% char. Additionally, 1-(benzyloxy)urea and two other lighter PAHs (benzylalcohol and benaldehyde) were detected in greater quantity in the FM17% char. The second replicate of the FM17% char sample exhibited a less diverse range of molecules, in particular by the absence of 1-(benzyloxy)urea and benzene (2,2-dichloroethyl). These molecules were present in the other three replicates. Only one chloroorganic molecule was detected—benzene (2,2-dichloroethyl)—discernible in three of the four samples and in very low quantities. Only one sulfur-substituted heterocyclic was detected—disulfide, bis(phenylmethyl)—present only in the FM7% char. The common fragment ion tropylium (*m*/*z* = 91) was present throughout and in all replicates at slightly above baseline levels.

## 4. Discussion

Many authors define highest treatment temperature (HTT) during pyrolysis as an important parameter for char characterization. This parameter does explain the higher volatile : FC ratio in the FM7% char caused by higher temperature exposure inside this zone of the gasifier. However, in some practical situations, as here with fixed-bed gasification char, the HTT concept has only limited relevance owing to the longer residence times and more varied reactor conditions *after* the pyrolysis zone. For example, in a downdraft gasifier, the post-pyrolysis char (on its gradual descent through the reactor) is further exposed to higher temperatures in the combustion zone where some char burns in the oxygenated environment. Following this HTT, the char is subjected to extended chemical and thermal treatment in the reduction zone. This invariably affects both its surface morphology (decarbonization and dehydrogenation through R1 and R2), likely extended FC aromatization, and continuous exposure to a throughput of gas-phase aromatics and aliphatic hydrocarbons from the upstream pyrolysis zone. A good way to highlight these secondary reactor effects on char is to compare these FM17% and FM7% char results with those from the updraft gasifier studied by San Miguel *et al.* [30]. Although the feedstock was identical and char elemental composition was similar, the FC : ash ratios were not, being two to four times greater in San Miguel's study. This difference is purely owing to the relative position of the reduction zone in the two gasifier designs. More specifically, it is the reduction zone's greater efficiency for consuming carbon via R1 and R2 in the downdraft gasifier in comparison to the updraft system as used in San Miguel's study. Hence, relatively lower ratios of FC : ash result from a downdraft gasifier as more carbon has been consumed internally. It is reasonable to speculate that such variable and non-trivial conditions would also occur in natural fires, particularly with increasing depth of combustible material and also where ventilation/air circulation is restricted. This illustrates why detailed descriptions and continuous monitoring of char production methods should be used as standard for future works that attempt to elucidate biochar properties. Further support for this comes from results of GC–MS and surface morphology experiments.

The differences in temperature observed in the gasifier reduction zone between FM7% and FM17% experiments were due to the parasitic energy demands of raising the temperature of H_2_O through its high values of latent (in the drying zone) and sensible enthalpy throughout. Internal moisture created lower temperatures and had the effect of suppressing the endothermic-reducing reactions (R1 and R2), resulting in lower CO at lower temperature operation. This also partly explains the higher CO_2_ concentrations in producer gas from the FM17% run (figure 3). The higher H_2_ concentration produced from gasification of FM17% cannot however be explained in this way, but it can be adequately described as being a function of the mildly exothermic water gas shift reaction (R3). By Le Chatelier's principle, R3 begins to favour its reverse direction as temperature increases. This led to higher H_2_ along with the lower concentration of CO and higher concentrations of CO_2_ with FM17% gasification. Of note is that some other (industrial) types of gasifier, such as fluidized bed and entrained flow, have quite different configurations which permit the control of air and steam-to-biomass ratio. In fixed-bed gasifiers, of the type used in this study, these variables cannot be adjusted.

The slightly higher pressure drop across the reactor observed with FM7% would have tended to inhibit gas transfer flow. This would consequently decrease access to char surface sites, and impair heat transfer therefore reducing chemical reaction rates. That no such adverse effect was observed evidences that the small differences in reactor pressure were insignificant in this regard.

Both char samples came within the biochar standard recommendations for elemental composition of H : C < 0.7 [43,47]. Such low H : C ratios have previously been shown to evidence char FC aromaticity [14], hence creating beneficial soil longevity and nutrient retention. Although the elemental analyses showed a H : C ratio at the lowest end of the biochar range (figure 4), with reference to other studies, the O : C ratio would be expected to be lower than actually observed. It can be seen with the GC–MS results (table 5) that the extractable hydrocarbons still contained many oxygenated functional groups, which may also be related to the high values of elemental O : C. The partial oxidation nature of gasification may account for this, such that less internal feedstock/char oxygen was consumed. It is worth noting however that the FM7% chars plot very close to the expected range, and that there was a much greater variation in both C and O elemental composition with the FM17% char. This suggests that the relatively high O : C values found in the FM17% char may be due in part to random error. Standard deviation values were much higher in elemental analyses of the FM17% char (table 3). These errors do not however correlate with any increased reactor instability (table 2).

The European Biochar Certificate suggests that there should be no more than 20% fluctuation in reactor temperature during char production, with the recommendation waived for small systems producing 20 tonnes p.a. [43]. This criterion was met, as the oscillations here were only 3% from the mean. As the variations in surface morphology and PAH content between the two samples was a significant finding of this study, a revision of the guidance notes, particularly with gasifier-derived biochar, seems appropriate. Although surface morphology does not form part of the EBC guideline, its potential influence on biochar efficacy is widely recognized [1,11,14]. Some previous studies have suggested that char surface area increases to a maximum at *ca* 600 ≤ °C ≤ 750, then declines significantly with increasing temperature [1,14,52]. Such a phenomenon was not observed here, and it is possible that these maxima may be attributable to unreported variations in previous char production methods.

The high surface area of both char samples and its inverse relation to increased feedstock moisture can again be explained by the reduction zone where char surface reactions remove carbon via endothermic R1 and R2. The downdraft gasifier chars from this study had 1 × 10^3^ times higher micropore volume and micropore area than the pine woodchip char produced in the updraft gasifier by San Miguel *et al.* [30]. Indeed, the values for micropore volume (correlated with BET surface area) were in the range of activated carbons produced, using a variety of methods [14]. This is not surprising considering that the reduction zone conditions in a downdraft gasifier—long residence time and exposure to a CO_2_ rich gas—are analogous to ‘activation’ processes used to increase the porosity and surface area of carbon [46]. The higher surface area and greater micropore volume in the FM7% biochar may account for the higher total PAHs detected in this sample owing to the increased capacity for PAH adsorption.

That no extractable PAHs with greater than four rings were detected in this study supports the gasifier engineering model of tar formation which predicts that evenly distributed and moderately high temperature inside the reactor would form tertiary tars but not to the extent where the heaviest GC-detectable PAH molecules were synthesized [16,20]. This hypothesis would then also predict that a greater quantity of low molecular weight PAHs and aliphatic hydrocarbons be detected in the FM17% char (owing to the lower temperatures), and that these molecules would be in lower concentration in the FM7% char extractate (owing to higher temperatures). This was observed. For hydrocarbons of molecular weight from naphthalene and above, all but two of the ubiquitous compounds were detected in greater concentration with the FM7% feedstock. Furthermore, the three molecules lighter than naphthalene: benzaldehyde, benzylalcohol and 1-(benzyloxy)urea were found in greater concentration in the FM17% sample (excepting that each of these molecules is absent in one of the three replicates).

Naphthalene is frequently reported as the commonest biochar PAH molecule detected by GC–MS, comprising up to 95%, and on average 40%, of the total Σ16EPA PAHs [18]. This study corroborates these findings as the naphthalene fraction was also between 40% and 60% of Σ16EPA PAH. It is interesting to note that two of the previous gasification biochar PAH studies identified naphthalene fractions at 7% [6] and 6% [30]. Both these studies also reported much higher concentrations of heavier tertiary tar molecules inferring, as described above, that these had been produced in a reactor operating at higher temperature and perhaps also relatively longer residence time.

Nitrogen enrichment from combustion has previously been reported [53,54], and this may explain the occurrence of 9H-carbazole, 9-ethyl; benzenemethanamine, *N*-(phenylmethylene); and 1-(benzyloxy)urea. However, it is also possible that some or all of these may be remnants of original plant functionality. This latter conjecture appears most relevant for 1-(benzyloxy)urea, which was detected in much higher quantity in the FM17% char (albeit in only one replicate). It had been exposed to less intense heat during gasification, so had been less subject to possible thermal decomposition. Urea is found in plants, where it is believed to be used as a nitrogen store, although the mechanisms of its accumulation and transport are not fully understood [55]. With regard to chlorinated hydrocarbons, previous literature to explore their presence in gasification and pyrolysis char is very scarce, with only one study acknowledged in the review by Buscheli *et al.* [18]. The absence of benzene (2,2-dichloroethyl) in one of the char extractate replicates and the closeness of their concentration values in the other three limits any further discussion.

To assess whether the PAHs in biochar could pose a danger to health and the environment, limit values have recently been proposed [43,47]. One suggests a range of maximum threshold values from 3 ≤ µg g^−1^ ≤ 300 Σ16EPA PAHs, derived from a selection of different jurisdictions (Europe and Australia) [47]. It was recommended to the author that although the full range represents a guide, concentrations within the higher limit value would be deemed sufficient (S Scotti 2015 Personal communication via e-mail, 24 November 2015). Both chars from this study would therefore be within this safe threshold. The second guideline defines two grades of biochar and the samples from this study would fall well outside the advised range of 12 (basic grade) and 4 µg g^−1^ (premium grade) [43]. This second guideline admits that the risk of PAH contamination is ‘…considered to be low, even if higher thresholds would be taken into account’. The reason for this uncertainty concerns a lack of definite proof about the extent of PAH bioavailability and whether any subsequent adverse effects may ensue. Soil-based tests have previously shown an equal number of positive to negative results [4–6]. The *terra preta do indio,* for example, has been dated at up to 7000 years old, and PAHs in char have been found to remain over geological time periods: charcoal from forest fire deposits in Triassic and Permo-Triassic boundary layers have PAH contents in the same range as those from recently produced pyrolysis [18]. To put this in greater context, urban and rural soils in the UK have concentrations of Σ22PAHs in the range of 0.04 ≤ µg g^−1^ ≤ 167, with the average being 2.2 µg g^−1^ [56]. Comparable values for this same set of hydrocarbons (many of which are on the Σ16EPA PAH list, although naphthalene is excluded) from these experiments are 27 ≤ µg g^−1^ ≤ 123, so again within the range, although above average. The 1.5 l volumes created per daily operation of this gasifier, assuming 300 days operation per year gives an annual output of char of 0.45 m^3^. If this is applied to a hectare at a recognized depth of 30 cm [29], it would constitute just 0.15% of the soil volume, or 0.002% on a mass basis (assuming a soil density of 1.4 g cm^−3^).

## 5. Conclusion

To better understand the relationship between production conditions and key biochar properties, a study was undertaken using a modern small-scale downdraft gasifier. Char was produced, while reactor conditions of temperature (either side of the char bed reduction zone), pressure and product gas output were closely monitored. Such gasifiers have a growing niche market for the provision of off-grid sustainable energy, producing useful amounts of wood char as a by-product. No previous assessments on how suitable this by-product is as biochar have been found.

Pine wood chip was used as gasifier feedstock and therefore biochar progenitor material. By setting its moisture content at two values: 17% (FM17%) and 7% (FM7%), this was the experimental variable. Such values of feedstock moisture are achievable by passive drying and relate directly to practical applications. Using FM17%, a lower reduction zone temperature was maintained in comparison with the drier feedstock (FM7%), with average values differing by 65°C at the top of the reduction zone and 49°C at the bottom of the reduction zone over a 6 h run. Significant differences in gas composition and calorific value occurred as a consequence. This had a significant effect on the biochar also, with a 62% difference in surface area: 749 (FM7%) and 467 m^2 ^g^−1^ (FM17%). Micropore volume and area showed equally significant differences, being 67% and 62% smaller in the FM17% char. These surface properties are similar to those of activated carbon and believed to be important for biochar functionality.

A clear dependence was also observed between feedstock moisture (hence operational temperature) and extractable PAHs. A greater total concentration of PAHs was detected with the FM7% biochar (187 µg g^−1^ ± 18 Σ16EPA PAH) than for the FM17% biochar (89 ± 19 µg g^−1^ Σ16EPA PAH). Many of the heavier PAHs identified as priority pollutants were not detected, evidencing a stable and moderately high gasification temperature during char production. Of particular note were the differences in acenaphthene and anthracene, with concentration ratios of 22 : 1 and 130 : 1 (FM7% : FM17%). Causes for these differences are explained by gasification thermochemical theory. Many nitrogen- and oxygen-substituted PAHs were observed in both biochar samples, and evidence of a trend appeared with lighter PAHs and two aliphatic compounds being either decreased in concentration or totally absent in the FM17% char. This is explained by tertiary PAH molecules being synthesized from primary and secondary biomass tars as a function of temperature and contact time.

This research has highlighted the importance of detailed monitoring and reporting of reactor conditions for future biochar studies. Data on feedstock moisture content or monitored operational parameters have hitherto been overlooked. The marked differences in biochar properties observed as a function of very small (49 ≤ °C ≤ 65) variations in production temperature indicate that any future study on biochar production methods should include as standard a more comprehensive scrutiny of production methods.

## Supplementary Material

Gasifier Data

## Supplementary Material

Toluene Extracted GCMS

## Supplementary Material

Biochar Volume Produced

## Supplementary Material

Ultimate, Proximate, and Feedstock Moisture Data

## Supplementary Material

Combined BET Data

## Data accessibility

The datasets supporting this article have been uploaded as supplementary material: Gasifier Data.xls; Ultimate, Proximate and Feedstock Moisture Data.xls; Biochar Volume Produced.xls; Combined BET Data.xls; Toluene Extracted GCMS.xls.
